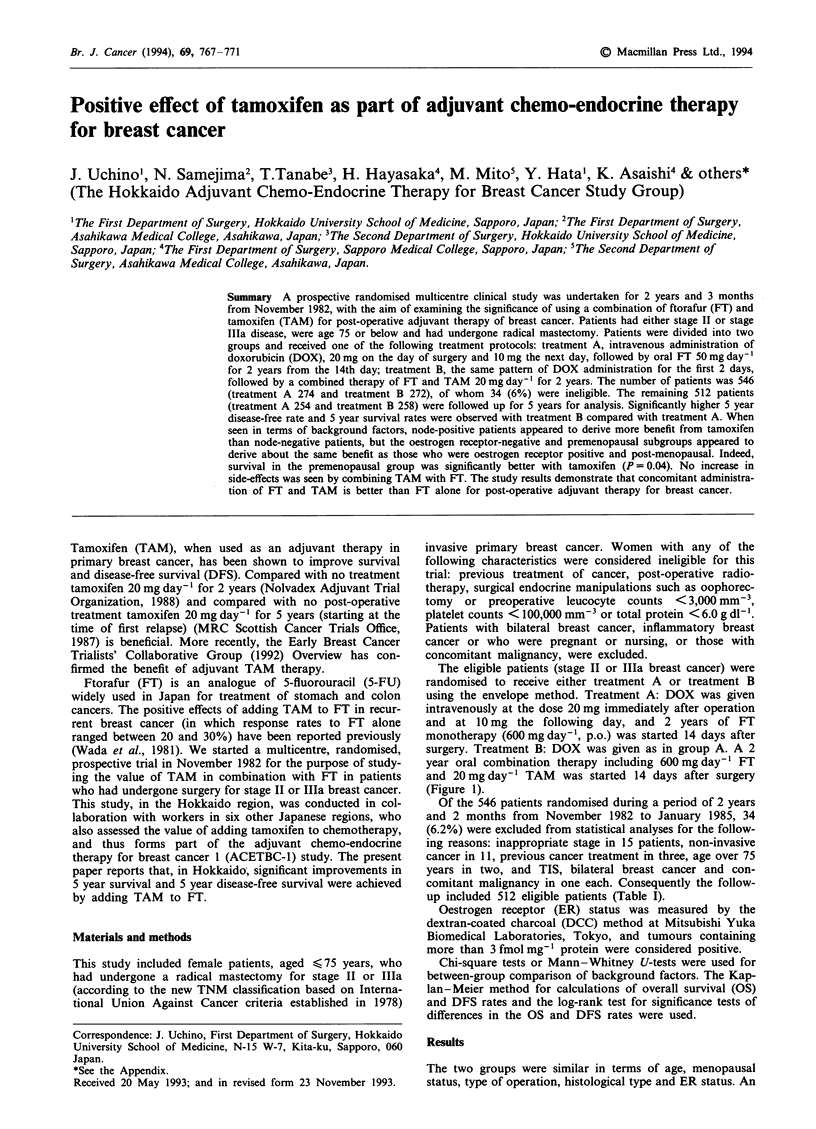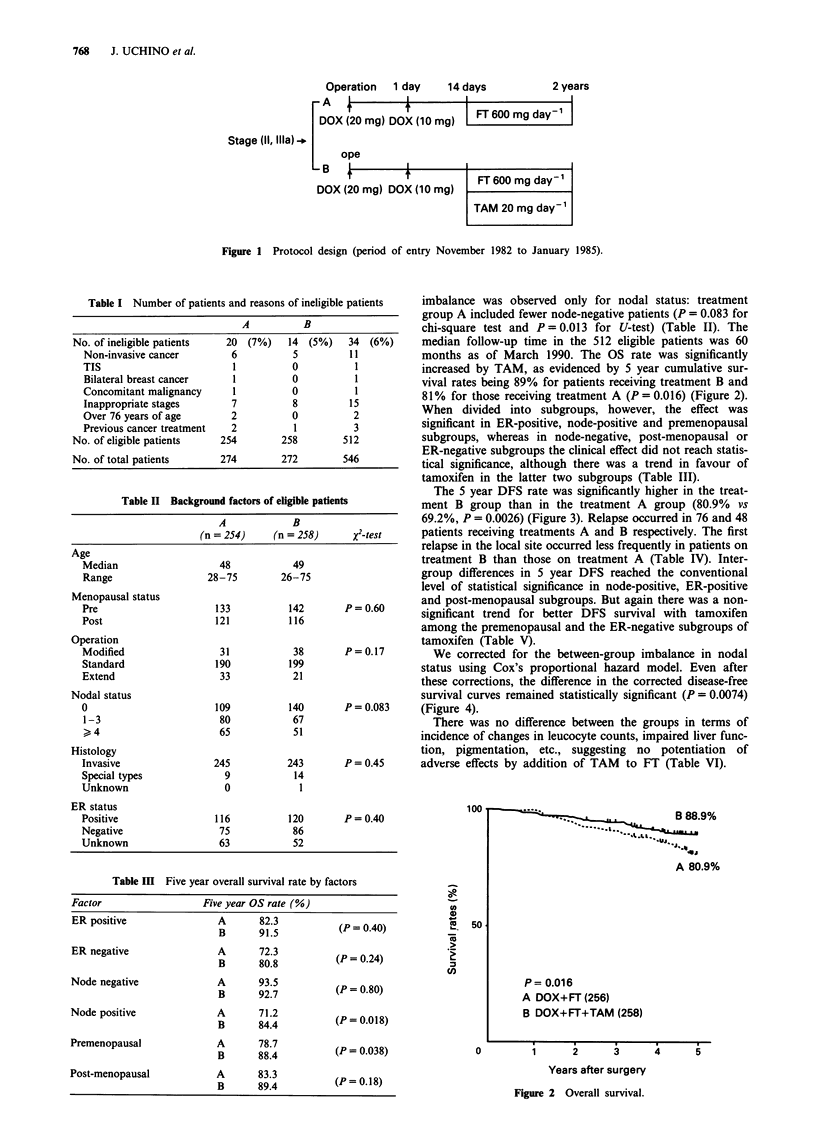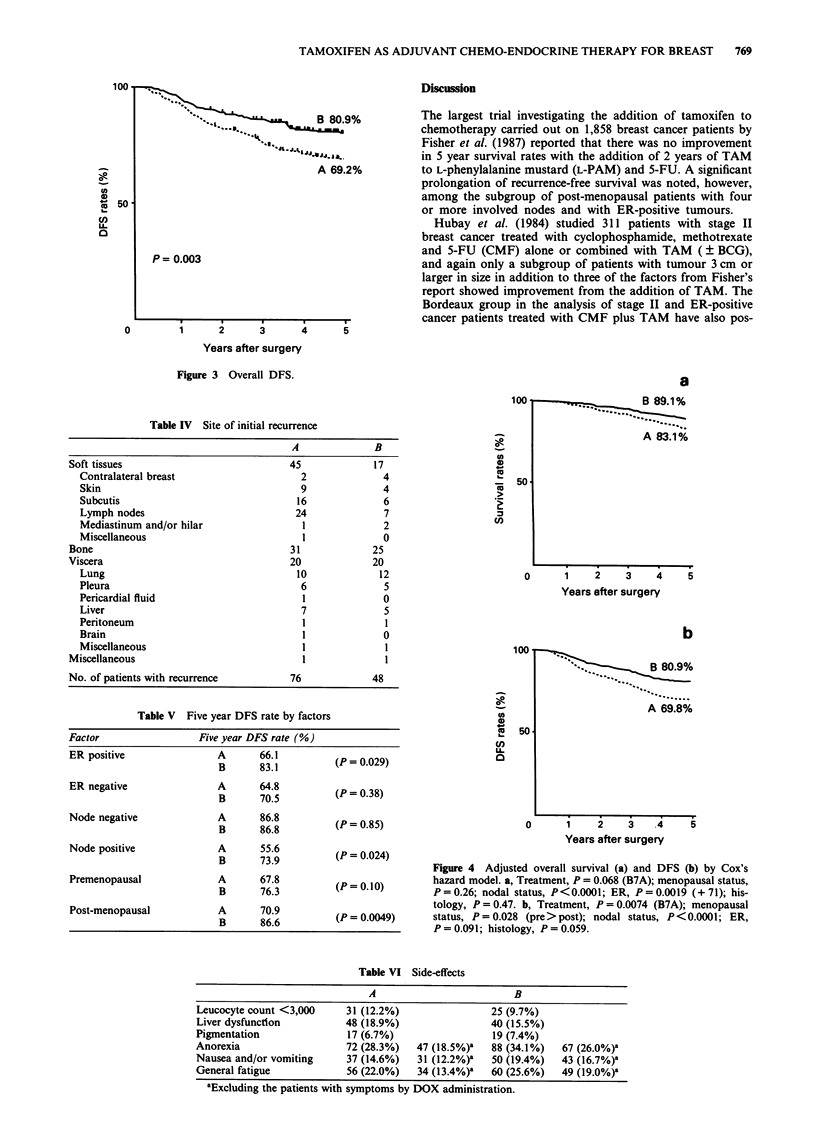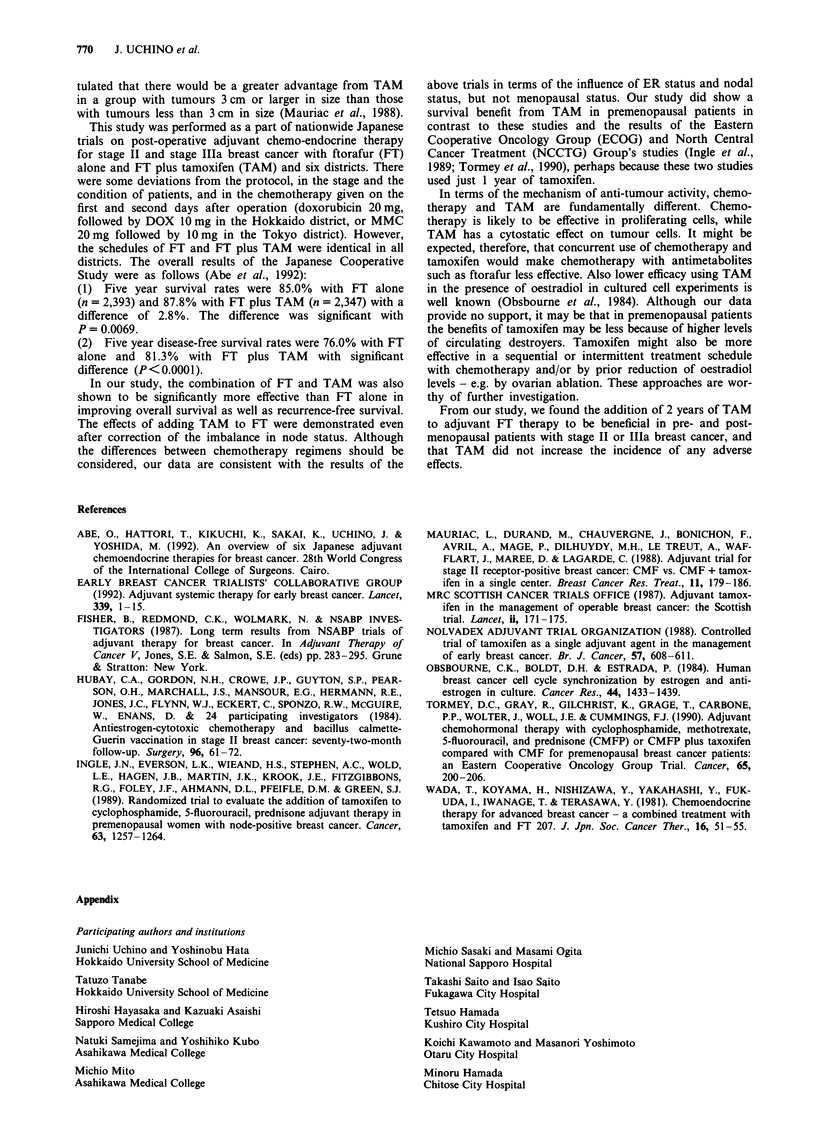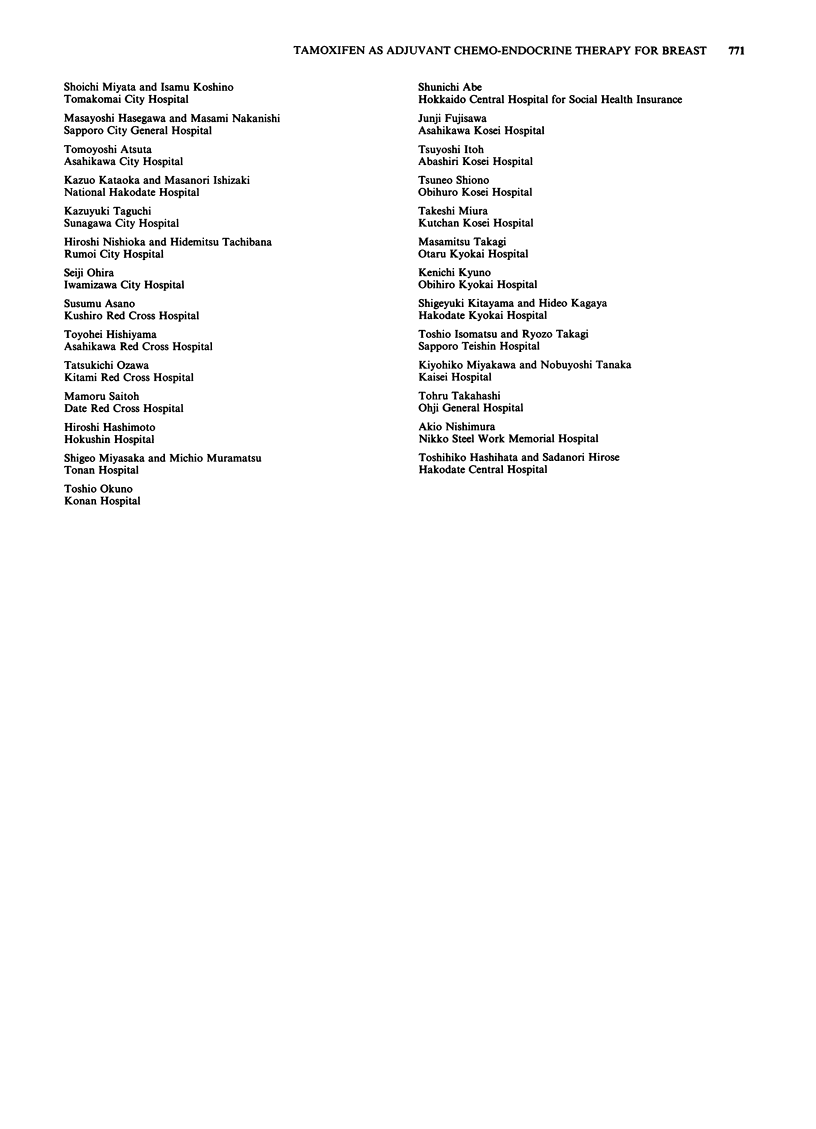# Positive effect of tamoxifen as part of adjuvant chemo-endocrine therapy for breast cancer. Hokkaido Adjuvant Chemo-Endocrine Therapy for Breast Cancer Study Group.

**DOI:** 10.1038/bjc.1994.145

**Published:** 1994-04

**Authors:** J. Uchino, N. Samejima, T. Tanabe, H. Hayasaka, M. Mito, Y. Hata, K. Asaishi

**Affiliations:** First Department of Surgery, Hokkaido University School of Medicine, Sapporo, Japan.

## Abstract

A prospective randomised multicentre clinical study was undertaken for 2 years and 3 months from November 1982, with the aim of examining the significance of using a combination of ftorafur (FT) and tamoxifen (TAM) for post-operative adjuvant therapy of breast cancer. Patients had either stage II or stage IIIa disease, were age 75 or below and had undergone radical mastectomy. Patients were divided into two groups and received one of the following treatment protocols: treatment A, intravenous administration of doxorubicin (DOX), 20 mg on the day of surgery and 10 mg the next day, followed by oral FT 50 mg day-1 for 2 years from the 14th day; treatment B, the same pattern of DOX administration for the first 2 days, followed by a combined therapy of FT and TAM 20 mg day-1 for 2 years. The number of patients was 546 (treatment A 274 and treatment B 272), of whom 34 (6%) were ineligible. The remaining 512 patients (treatment A 254 and treatment B 258) were followed up for 5 years for analysis. Significantly higher 5 year disease-free rate and 5 year survival rates were observed with treatment B compared with treatment A. When seen in terms of background factors, node-positive patients appeared to derive more benefit from tamoxifen than node-negative patients, but the oestrogen receptor-negative and premenopausal subgroups appeared to derive about the same benefit as those who were oestrogen receptor positive and post-menopausal. Indeed, survival in the premenopausal group was significantly better with tamoxifen (P = 0.04). No increase in side-effects was seen by combining TAM with FT. The study results demonstrate that concomitant administration of FT and TAM is better than FT alone for post-operative adjuvant therapy for breast cancer.


Br. J. Cancer (1994), 69, 767-771
© Macmillan Press Ltd., 1994

### Positive effect of tamoxifen as part of adjuvant chemo-endocrine therapy for breast cancer

J. Uchino[1], N. Samejima[2], T.Tanabe[3], H. Hayasaka[4], M. Mito[5], Y. Hata[1], K. Asaishi[4] & others*
(The Hokkaido Adjuvant Chemo-Endocrine Therapy for Breast Cancer Study Group)

[1]The First Department of Surgery, Hokkaido University School of Medicine, Sapporo, Japan; [2]The First Department of Surgery, Asahikawa Medical College, Asahikawa, Japan; [3]The Second Department of Surgery, Hokkaido University School of Medicine, Sapporo, Japan; [4]The First Department of Surgery, Sapporo Medical College, Sapporo, Japan; [5]The Second Department of Surgery, Asahikawa Medical College, Asahikawa, Japan.

**Summary** A prospective randomised multicentre clinical study was undertaken for 2 years and 3 months from November 1982, with the aim of examining the significance of using a combination of ftorafur (FT) and tamoxifen (TAM) for post-operative adjuvant therapy of breast cancer. Patients had either stage II or stage IIIa disease, were age 75 or below and had undergone radical mastectomy. Patients were divided into two groups and received one of the following treatment protocols: treatment A, intravenous administration of doxorubicin (DOX), 20 mg on the day of surgery and 10 mg the next day, followed by oral FT 50 mg day$^{-1}$ for 2 years from the 14th day; treatment B, the same pattern of DOX administration for the first 2 days, followed by a combined therapy of FT and TAM 20 mg day$^{-1}$ for 2 years. The number of patients was 546 (treatment A 274 and treatment B 272), of whom 34 (6%) were ineligible. The remaining 512 patients (treatment A 254 and treatment B 258) were followed up for 5 years for analysis. Significantly higher 5 year disease-free rate and 5 year survival rates were observed with treatment B compared with treatment A. When seen in terms of background factors, node-positive patients appeared to derive more benefit from tamoxifen than node-negative patients, but the oestrogen receptor-negative and premenopausal subgroups appeared to derive about the same benefit as those who were oestrogen receptor positive and post-menopausal. Indeed, survival in the premenopausal group was significantly better with tamoxifen ($P = 0.04$). No increase in side-effects was seen by combining TAM with FT. The study results demonstrate that concomitant administration of FT and TAM is better than FT alone for post-operative adjuvant therapy for breast cancer.

Tamoxifen (TAM), when used as an adjuvant therapy in primary breast cancer, has been shown to improve survival and disease-free survival (DFS). Compared with no treatment tamoxifen 20 mg day$^{-1}$ for 2 years (Nolvadex Adjuvant Trial Organization, 1988) and compared with no post-operative treatment tamoxifen 20 mg day$^{-1}$ for 5 years (starting at the time of first relapse) (MRC Scottish Cancer Trials Office, 1987) is beneficial. More recently, the Early Breast Cancer Trialists' Collaborative Group (1992) Overview has confirmed the benefit of adjuvant TAM therapy.

Ftorafur (FT) is an analogue of 5-fluorouracil (5-FU) widely used in Japan for treatment of stomach and colon cancers. The positive effects of adding TAM to FT in recurrent breast cancer (in which response rates to FT alone ranged between 20 and 30%) have been reported previously (Wada *et al.*, 1981). We started a multicentre, randomised, prospective trial in November 1982 for the purpose of studying the value of TAM in combination with FT in patients who had undergone surgery for stage II or IIIa breast cancer. This study, in the Hokkaido region, was conducted in collaboration with workers in six other Japanese regions, who also assessed the value of adding tamoxifen to chemotherapy, and thus forms part of the adjuvant chemo-endocrine therapy for breast cancer 1 (ACETBC-1) study. The present paper reports that, in Hokkaido, significant improvements in 5 year survival and 5 year disease-free survival were achieved by adding TAM to FT.

### Materials and methods

This study included female patients, aged ⩽75 years, who had undergone a radical mastectomy for stage II or IIIa (according to the new TNM classification based on International Union Against Cancer criteria established in 1978) invasive primary breast cancer. Women with any of the following characteristics were considered ineligible for this trial: previous treatment of cancer, post-operative radiotherapy, surgical endocrine manipulations such as oophorectomy or preoperative leucocyte counts $<3,000\ mm^{-3}$, platelet counts $<100,000\ mm^{-3}$ or total protein $<6.0\ g\ dl^{-1}$. Patients with bilateral breast cancer, inflammatory breast cancer or who were pregnant or nursing, or those with concomitant malignancy, were excluded.

The eligible patients (stage II or IIIa breast cancer) were randomised to receive either treatment A or treatment B using the envelope method. Treatment A: DOX was given intravenously at the dose 20 mg immediately after operation and at 10 mg the following day, and 2 years of FT monotherapy (600 mg day$^{-1}$, p.o.) was started 14 days after surgery. Treatment B: DOX was given as in group A. A 2 year oral combination therapy including 600 mg day$^{-1}$ FT and 20 mg day$^{-1}$ TAM was started 14 days after surgery (Figure 1).

Of the 546 patients randomised during a period of 2 years and 2 months from November 1982 to January 1985, 34 (6.2%) were excluded from statistical analyses for the following reasons: inappropriate stage in 15 patients, non-invasive cancer in 11, previous cancer treatment in three, age over 75 years in two, and TIS, bilateral breast cancer and concomitant malignancy in one each. Consequently the follow-up included 512 eligible patients (Table I).

Oestrogen receptor (ER) status was measured by the dextran-coated charcoal (DCC) method at Mitsubishi Yuka Biomedical Laboratories, Tokyo, and tumours containing more than 3 fmol mg$^{-1}$ protein were considered positive.

Chi-square tests or Mann–Whitney $U$-tests were used for between-group comparison of background factors. The Kaplan–Meier method for calculations of overall survival (OS) and DFS rates and the log-rank test for significance tests of differences in the OS and DFS rates were used.

### Results

The two groups were similar in terms of age, menopausal status, type of operation, histological type and ER status. An

Correspondence: J. Uchino, First Department of Surgery, Hokkaido University School of Medicine, N-15 W-7, Kita-ku, Sapporo, 060 Japan.
*See the Appendix.
Received 20 May 1993; and in revised form 23 November 1993.

**Figure 1** Protocol design (period of entry November 1982 to January 1985).

**Table I** Number of patients and reasons of ineligible patients

| | *A* | *B* | |
|---|---|---|---|
| No. of ineligible patients | 20 (7%) | 14 (5%) | 34 (6%) |
| Non-invasive cancer | 6 | 5 | 11 |
| TIS | 1 | 0 | 1 |
| Bilateral breast cancer | 1 | 0 | 1 |
| Concomitant malignancy | 1 | 0 | 1 |
| Inappropriate stages | 7 | 8 | 15 |
| Over 76 years of age | 2 | 0 | 2 |
| Previous cancer treatment | 2 | 1 | 3 |
| No. of eligible patients | 254 | 258 | 512 |
| No. of total patients | 274 | 272 | 546 |

**Table II Background factors of eligible patients**

| | *A* (n = 254) | *B* (n = 258) | $\chi^2$-*test* |
|---|---|---|---|
| Age | | | |
| Median | 48 | 49 | |
| Range | 28–75 | 26–75 | |
| Menopausal status | | | |
| Pre | 133 | 142 | $P = 0.60$ |
| Post | 121 | 116 | |
| Operation | | | |
| Modified | 31 | 38 | $P = 0.17$ |
| Standard | 190 | 199 | |
| Extend | 33 | 21 | |
| Nodal status | | | |
| 0 | 109 | 140 | $P = 0.083$ |
| 1–3 | 80 | 67 | |
| ⩾4 | 65 | 51 | |
| Histology | | | |
| Invasive | 245 | 243 | $P = 0.45$ |
| Special types | 9 | 14 | |
| Unknown | 0 | 1 | |
| ER status | | | |
| Positive | 116 | 120 | $P = 0.40$ |
| Negative | 75 | 86 | |
| Unknown | 63 | 52 | |

**Table III** Five year overall survival rate by factors

| *Factor* | *Five year OS rate (%)* | | |
|---|---|---|---|
| ER positive | A | 82.3 | ($P = 0.40$) |
| | B | 91.5 | |
| ER negative | A | 72.3 | ($P = 0.24$) |
| | B | 80.8 | |
| Node negative | A | 93.5 | ($P = 0.80$) |
| | B | 92.7 | |
| Node positive | A | 71.2 | ($P = 0.018$) |
| | B | 84.4 | |
| Premenopausal | A | 78.7 | ($P = 0.038$) |
| | B | 88.4 | |
| Post-menopausal | A | 83.3 | ($P = 0.18$) |
| | B | 89.4 | |

imbalance was observed only for nodal status: treatment group A included fewer node-negative patients ($P = 0.083$ for chi-square test and $P = 0.013$ for *U*-test) (Table II). The median follow-up time in the 512 eligible patients was 60 months as of March 1990. The OS rate was significantly increased by TAM, as evidenced by 5 year cumulative survival rates being 89% for patients receiving treatment B and 81% for those receiving treatment A ($P = 0.016$) (Figure 2). When divided into subgroups, however, the effect was significant in ER-positive, node-positive and premenopausal subgroups, whereas in node-negative, post-menopausal or ER-negative subgroups the clinical effect did not reach statistical significance, although there was a trend in favour of tamoxifen in the latter two subgroups (Table III).

The 5 year DFS rate was significantly higher in the treatment B group than in the treatment A group (80.9% *vs* 69.2%, $P = 0.0026$) (Figure 3). Relapse occurred in 76 and 48 patients receiving treatments A and B respectively. The first relapse in the local site occurred less frequently in patients on treatment B than those on treatment A (Table IV). Intergroup differences in 5 year DFS reached the conventional level of statistical significance in node-positive, ER-positive and post-menopausal subgroups. But again there was a non-significant trend for better DFS survival with tamoxifen among the premenopausal and the ER-negative subgroups of tamoxifen (Table V).

We corrected for the between-group imbalance in nodal status using Cox's proportional hazard model. Even after these corrections, the difference in the corrected disease-free survival curves remained statistically significant ($P = 0.0074$) (Figure 4).

There was no difference between the groups in terms of incidence of changes in leucocyte counts, impaired liver function, pigmentation, etc., suggesting no potentiation of adverse effects by addition of TAM to FT (Table VI).

**Figure 2** Overall survival.

**Figure 3** Overall DFS.

**Table IV** Site of initial recurrence

| | A | B |
|---|---|---|
| Soft tissues | 45 | 17 |
| Contralateral breast | 2 | 4 |
| Skin | 9 | 4 |
| Subcutis | 16 | 6 |
| Lymph nodes | 24 | 7 |
| Mediastinum and/or hilar | 1 | 2 |
| Miscellaneous | 1 | 0 |
| Bone | 31 | 25 |
| Viscera | 20 | 20 |
| Lung | 10 | 12 |
| Pleura | 6 | 5 |
| Pericardial fluid | 1 | 0 |
| Liver | 7 | 5 |
| Peritoneum | 1 | 1 |
| Brain | 1 | 0 |
| Miscellaneous | 1 | 1 |
| Miscellaneous | 1 | 1 |
| No. of patients with recurrence | 76 | 48 |

**Table V** Five year DFS rate by factors

| Factor | Five year DFS rate (%) | | |
|---|---|---|---|
| ER positive | A | 66.1 | ($P = 0.029$) |
| | B | 83.1 | |
| ER negative | A | 64.8 | ($P = 0.38$) |
| | B | 70.5 | |
| Node negative | A | 86.8 | ($P = 0.85$) |
| | B | 86.8 | |
| Node positive | A | 55.6 | ($P = 0.024$) |
| | B | 73.9 | |
| Premenopausal | A | 67.8 | ($P = 0.10$) |
| | B | 76.3 | |
| Post-menopausal | A | 70.9 | ($P = 0.0049$) |
| | B | 86.6 | |

### Discussion

The largest trial investigating the addition of tamoxifen to chemotherapy carried out on 1,858 breast cancer patients by Fisher *et al.* (1987) reported that there was no improvement in 5 year survival rates with the addition of 2 years of TAM to L-phenylalanine mustard (L-PAM) and 5-FU. A significant prolongation of recurrence-free survival was noted, however, among the subgroup of post-menopausal patients with four or more involved nodes and with ER-positive tumours.

Hubay *et al.* (1984) studied 311 patients with stage II breast cancer treated with cyclophosphamide, methotrexate and 5-FU (CMF) alone or combined with TAM ($\pm$ BCG), and again only a subgroup of patients with tumour 3 cm or larger in size in addition to three of the factors from Fisher's report showed improvement from the addition of TAM. The Bordeaux group in the analysis of stage II and ER-positive cancer patients treated with CMF plus TAM have also pos-

**Figure 4** Adjusted overall survival (**a**) and DFS (**b**) by Cox's hazard model. **a**, Treatment, $P = 0.068$ (B7A); menopausal status, $P = 0.26$; nodal status, $P < 0.0001$; ER, $P = 0.0019$ (+71); histology, $P = 0.47$. **b**, Treatment, $P = 0.0074$ (B7A); menopausal status, $P = 0.028$ (pre>post); nodal status, $P < 0.0001$; ER, $P = 0.091$; histology, $P = 0.059$.

**Table VI** Side-effects

| | A | | B | |
|---|---|---|---|---|
| Leucocyte count <3,000 | 31 (12.2%) | | 25 (9.7%) | |
| Liver dysfunction | 48 (18.9%) | | 40 (15.5%) | |
| Pigmentation | 17 (6.7%) | | 19 (7.4%) | |
| Anorexia | 72 (28.3%) | 47 (18.5%)[a] | 88 (34.1%) | 67 (26.0%)[a] |
| Nausea and/or vomiting | 37 (14.6%) | 31 (12.2%)[a] | 50 (19.4%) | 43 (16.7%)[a] |
| General fatigue | 56 (22.0%) | 34 (13.4%)[a] | 60 (25.6%) | 49 (19.0%)[a] |

[a]Excluding the patients with symptoms by DOX administration.

tulated that there would be a greater advantage from TAM in a group with tumours 3 cm or larger in size than those with tumours less than 3 cm in size (Mauriac et al., 1988).

This study was performed as a part of nationwide Japanese trials on post-operative adjuvant chemo-endocrine therapy for stage II and stage IIIa breast cancer with ftorafur (FT) alone and FT plus tamoxifen (TAM) and six districts. There were some deviations from the protocol, in the stage and the condition of patients, and in the chemotherapy given on the first and second days after operation (doxorubicin 20 mg, followed by DOX 10 mg in the Hokkaido district, or MMC 20 mg followed by 10 mg in the Tokyo district). However, the schedules of FT and FT plus TAM were identical in all districts. The overall results of the Japanese Cooperative Study were as follows (Abe et al., 1992):

(1) Five year survival rates were 85.0% with FT alone ($n = 2,393$) and 87.8% with FT plus TAM ($n = 2,347$) with a difference of 2.8%. The difference was significant with $P = 0.0069$.

(2) Five year disease-free survival rates were 76.0% with FT alone and 81.3% with FT plus TAM with significant difference ($P < 0.0001$).

In our study, the combination of FT and TAM was also shown to be significantly more effective than FT alone in improving overall survival as well as recurrence-free survival. The effects of adding TAM to FT were demonstrated even after correction of the imbalance in node status. Although the differences between chemotherapy regimens should be considered, our data are consistent with the results of the above trials in terms of the influence of ER status and nodal status, but not menopausal status. Our study did show a survival benefit from TAM in premenopausal patients in contrast to these studies and the results of the Eastern Cooperative Oncology Group (ECOG) and North Central Cancer Treatment (NCCTG) Group's studies (Ingle et al., 1989; Tormey et al., 1990), perhaps because these two studies used just 1 year of tamoxifen.

In terms of the mechanism of anti-tumour activity, chemotherapy and TAM are fundamentally different. Chemotherapy is likely to be effective in proliferating cells, while TAM has a cytostatic effect on tumour cells. It might be expected, therefore, that concurrent use of chemotherapy and tamoxifen would make chemotherapy with antimetabolites such as ftorafur less effective. Also lower efficacy using TAM in the presence of oestradiol in cultured cell experiments is well known (Obsbourne et al., 1984). Although our data provide no support, it may be that in premenopausal patients the benefits of tamoxifen may be less because of higher levels of circulating destroyers. Tamoxifen might also be more effective in a sequential or intermittent treatment schedule with chemotherapy and/or by prior reduction of oestradiol levels – e.g. by ovarian ablation. These approaches are worthy of further investigation.

From our study, we found the addition of 2 years of TAM to adjuvant FT therapy to be beneficial in pre- and post-menopausal patients with stage II or IIIa breast cancer, and that TAM did not increase the incidence of any adverse effects.

### References

ABE, O., HATTORI, T., KIKUCHI, K., SAKAI, K., UCHINO, J. & YOSHIDA, M. (1992). An overview of six Japanese adjuvant chemoendocrine therapies for breast cancer. 28th World Congress of the International College of Surgeons. Cairo.

EARLY BREAST CANCER TRIALISTS' COLLABORATIVE GROUP (1992). Adjuvant systemic therapy for early breast cancer. *Lancet*, **339**, 1–15.

FISHER, B., REDMOND, C.K., WOLMARK, N. & NSABP INVESTIGATORS (1987). Long term results from NSABP trials of adjuvant therapy for breast cancer. In *Adjuvant Therapy of Cancer V*, Jones, S.E. & Salmon, S.E. (eds) pp. 283–295. Grune & Stratton: New York.

HUBAY, C.A., GORDON, N.H., CROWE, J.P., GUYTON, S.P., PEARSON, O.H., MARCHALL, J.S., MANSOUR, E.G., HERMANN, R.E., JONES, J.C., FLYNN, W.J., ECKERT, C., SPONZO, R.W., MCGUIRE, W., ENANS, D. & 24 participating investigators (1984). Antiestrogen-cytotoxic chemotherapy and bacillus calmette-Guerin vaccination in stage II breast cancer: seventy-two-month follow-up. *Surgery*, **96**, 61–72.

INGLE, J.N., EVERSON, L.K., WIEAND, H.S., STEPHEN, A.C., WOLD, L.E., HAGEN, J.B., MARTIN, J.K., KROOK, J.E., FITZGIBBONS, R.G., FOLEY, J.F., AHMANN, D.L., PFEIFLE, D.M. & GREEN, S.J. (1989). Randomized trial to evaluate the addition of tamoxifen to cyclophosphamide, 5-fluorouracil, prednisone adjuvant therapy in premenopausal women with node-positive breast cancer. *Cancer*, **63**, 1257–1264.

MAURIAC, L., DURAND, M., CHAUVERGNE, J., BONICHON, F., AVRIL, A., MAGE, P., DILHUYDY, M.H., LE TREUT, A., WAF-FLART, J., MAREE, D. & LAGARDE, C. (1988). Adjuvant trial for stage II receptor-positive breast cancer: CMF vs. CMF + tamoxifen in a single center. *Breast Cancer Res. Treat.*, **11**, 179–186.

MRC SCOTTISH CANCER TRIALS OFFICE (1987). Adjuvant tamoxifen in the management of operable breast cancer: the Scottish trial. *Lancet*, **ii**, 171–175.

NOLVADEX ADJUVANT TRIAL ORGANIZATION (1988). Controlled trial of tamoxifen as a single adjuvant agent in the management of early breast cancer. *Br. J. Cancer*, **57**, 608–611.

OBSBOURNE, C.K., BOLDT, D.H. & ESTRADA, P. (1984). Human breast cancer cell cycle synchronization by estrogen and anti-estrogen in culture. *Cancer Res.*, **44**, 1433–1439.

TORMEY, D.C., GRAY, R., GILCHRIST, K., GRAGE, T., CARBONE, P.P., WOLTER, J., WOLL, J.E. & CUMMINGS, F.J. (1990). Adjuvant chemohormonal therapy with cyclophosphamide, methotrexate, 5-fluorouracil, and prednisone (CMFP) or CMFP plus taxoxifen compared with CMF for premenopausal breast cancer patients: an Eastern Cooperative Oncology Group Trial. *Cancer*, **65**, 200–206.

WADA, T., KOYAMA, H., NISHIZAWA, Y., YAKAHASHI, Y., FUKUDA, I., IWANAGE, T. & TERASAWA, Y. (1981). Chemoendocrine therapy for advanced breast cancer – a combined treatment with tamoxifen and FT 207. *J. Jpn. Soc. Cancer Ther.*, **16**, 51–55.

### Appendix

*Participating authors and institutions*

Junichi Uchino and Yoshinobu Hata
Hokkaido University School of Medicine

Tatuzo Tanabe
Hokkaido University School of Medicine

Hiroshi Hayasaka and Kazuaki Asaishi
Sapporo Medical College

Natuki Samejima and Yoshihiko Kubo
Asahikawa Medical College

Michio Mito
Asahikawa Medical College

Michio Sasaki and Masami Ogita
National Sapporo Hospital

Takashi Saito and Isao Saito
Fukagawa City Hospital

Tetsuo Hamada
Kushiro City Hospital

Koichi Kawamoto and Masanori Yoshimoto
Otaru City Hospital

Minoru Hamada
Chitose City Hospital

Shoichi Miyata and Isamu Koshino
Tomakomai City Hospital

Masayoshi Hasegawa and Masami Nakanishi
Sapporo City General Hospital

Tomoyoshi Atsuta
Asahikawa City Hospital

Kazuo Kataoka and Masanori Ishizaki
National Hakodate Hospital

Kazuyuki Taguchi
Sunagawa City Hospital

Hiroshi Nishioka and Hidemitsu Tachibana
Rumoi City Hospital

Seiji Ohira
Iwamizawa City Hospital

Susumu Asano
Kushiro Red Cross Hospital

Toyohei Hishiyama
Asahikawa Red Cross Hospital

Tatsukichi Ozawa
Kitami Red Cross Hospital

Mamoru Saitoh
Date Red Cross Hospital

Hiroshi Hashimoto
Hokushin Hospital

Shigeo Miyasaka and Michio Muramatsu
Tonan Hospital

Toshio Okuno
Konan Hospital

Shunichi Abe
Hokkaido Central Hospital for Social Health Insurance

Junji Fujisawa
Asahikawa Kosei Hospital

Tsuyoshi Itoh
Abashiri Kosei Hospital

Tsuneo Shiono
Obihuro Kosei Hospital

Takeshi Miura
Kutchan Kosei Hospital

Masamitsu Takagi
Otaru Kyokai Hospital

Kenichi Kyuno
Obihiro Kyokai Hospital

Shigeyuki Kitayama and Hideo Kagaya
Hakodate Kyokai Hospital

Toshio Isomatsu and Ryozo Takagi
Sapporo Teishin Hospital

Kiyohiko Miyakawa and Nobuyoshi Tanaka
Kaisei Hospital

Tohru Takahashi
Ohji General Hospital

Akio Nishimura
Nikko Steel Work Memorial Hospital

Toshihiko Hashihata and Sadanori Hirose
Hakodate Central Hospital